# ACTION-EHR: Patient-Centric Blockchain-Based Electronic Health Record Data Management for Cancer Care

**DOI:** 10.2196/13598

**Published:** 2020-08-21

**Authors:** Alevtina Dubovitskaya, Furqan Baig, Zhigang Xu, Rohit Shukla, Pratik Sushil Zambani, Arun Swaminathan, Md Majid Jahangir, Khadija Chowdhry, Rahul Lachhani, Nitesh Idnani, Michael Schumacher, Karl Aberer, Scott D Stoller, Samuel Ryu, Fusheng Wang

**Affiliations:** 1 School of Information Technology Lucerne University of Applied Sciences and Arts Rotkreuz Switzerland; 2 Swisscom Zurich Switzerland; 3 Department of Computer Science Stony Brook University Stony Brook, NY United States; 4 Department of Radiation Oncology Stony Brook Medicine Stony Brook, NY United States; 5 Applied Intelligent Systems Lab University of Applied Sciences of Western Switzerland - Valais Sierre Switzerland; 6 Polytechnic University of Lausanne Lausanne Switzerland; 7 Department of Biomedical Informatics Stony Brook University Stony Brook, NY United States

**Keywords:** electronic health records, data sharing, blockchain, Hyperledger Fabric, privacy, security

## Abstract

**Background:**

With increased specialization of health care services and high levels of patient mobility, accessing health care services across multiple hospitals or clinics has become very common for diagnosis and treatment, particularly for patients with chronic diseases such as cancer. With informed knowledge of a patient’s history, physicians can make prompt clinical decisions for smarter, safer, and more efficient care. However, due to the privacy and high sensitivity of electronic health records (EHR), most EHR data sharing still happens through fax or mail due to the lack of systematic infrastructure support for secure, trustable health data sharing, which can also cause major delays in patient care.

**Objective:**

Our goal was to develop a system that will facilitate secure, trustable management, sharing, and aggregation of EHR data. Our patient-centric system allows patients to manage their own health records across multiple hospitals. The system will ensure patient privacy protection and guarantee security with respect to the requirements for health care data management, including the access control policy specified by the patient.

**Methods:**

We propose a permissioned blockchain-based system for EHR data sharing and integration. Each hospital will provide a blockchain node integrated with its own EHR system to form the blockchain network. A web-based interface will be used for patients and doctors to initiate EHR sharing transactions. We take a hybrid data management approach, where only management metadata will be stored on the chain. Actual EHR data, on the other hand, will be encrypted and stored off-chain in Health Insurance Portability and Accountability Act–compliant cloud-based storage. The system uses public key infrastructure–based asymmetric encryption and digital signatures to secure shared EHR data.

**Results:**

In collaboration with Stony Brook University Hospital, we developed ACTION-EHR, a system for patient-centric, blockchain-based EHR data sharing and management for patient care, in particular radiation treatment for cancer. The prototype was built on Hyperledger Fabric, an open-source, permissioned blockchain framework. Data sharing transactions were implemented using chaincode and exposed as representational state transfer application programming interfaces used for the web portal for patients and users. The HL7 Fast Healthcare Interoperability Resources standard was adopted to represent shared EHR data, making it easy to interface with hospital EHR systems and integrate a patient’s EHR data. We tested the system in a distributed environment at Stony Brook University using deidentified patient data.

**Conclusions:**

We studied and developed the critical technology components to enable patient-centric, blockchain-based EHR sharing to support cancer care. The prototype demonstrated the feasibility of our approach as well as some of the major challenges. The next step will be a pilot study with health care providers in both the United States and Switzerland. Our work provides an exemplar testbed to build next-generation EHR sharing infrastructures.

## Introduction

Timely sharing of electronic health records (EHR) across providers is essential for prompt medical care. For instance, transition and coordination of care for cancer patients are very common phenomena. A patient’s history of health, tests, diagnoses, and treatments provides necessary knowledge for physicians to make clinical decisions. Access to EHR history is also preferred by individual patients to support personal and family engagement with user-centric control of data sharing and access [1]. Historical EHR data can also empower predictive modeling to drive personalized medicine and improve health care quality through machine learning [2].

EHR data are highly private and sensitive. According to the Health Insurance Portability and Accountability Act (HIPAA) [3], a patient has the right over his health information and can set rules and limits on who can access and receive health information. In current practice, if a patient needs to transfer his clinical data from one hospital to another, he is typically required to sign paper-based consent that specifies what type of data will be shared and the information about the recipient. EHR data sharing is mostly still a tedious manual process through fax or mail and often takes days or even months for the records to become available. This is mainly due to a lack of systematic infrastructure support for secure and trustable EHR data sharing, which may also incur major delays for patient care.

Ecosystems for health information exchange (HIE) aim to ensure that patient data from EHR are securely, efficiently, and accurately shared nationwide. However, HIEs have limited adoption, and many regional networks are still isolated [4]. Furthermore, the current system lacks standard architecture, resulting in a failure to ensure proper security and access control for patients once data are shared.

HIEs are generally designed as a single, fully trusted entity that is solely responsible for managing and storing EHR data from multiple participating hospitals. While a centralized system may be easier to manage, it suffers from a single point of failure and may prove to be a performance bottleneck for real-world deployment. In addition, a centralized authority with access to sensitive health information has proven to have more security and privacy concerns from end users. The experience with GoogleHealth wallet [5], for example, has shown that patients are concerned about their privacy and aware of the potential risk that their sensitive data might be misused. Alternatively, all the data can be stored and managed in the encrypted form (eg, using homomorphic encryption) for increased security and privacy. However, it requires large amounts of memory and extensive computations [6] that can be prohibitive for a hospital environment. Partial data encryption can improve the efficiency of such methods [7]; however, in medical settings, there might be a need to access and analyze all historical data and images (that lack any encryption) for better health care decisions.

Blockchain technologies have recently emerged with tremendous momentum based on the success of the Bitcoin cryptocurrency [8]. Blockchain uses a distributed ledger to provide a shared, immutable, and transparent history of all the actions that have happened to all the participants of the network. It enables a new generation of transactional applications that establish trust, accountability, and transparency. Blockchain, in particular permissioned blockchain technology, makes it possible for a user to have complete control of data and privacy without a central point of control; thus, it is highly cost-effective and efficient for building applications for sharing EHR data [9-15].

In this paper, we propose ACTION-EHR, a patient-centric, secure, trustable EHR data sharing framework with permissioned blockchain framework that can not only accelerate the data sharing process but also enable patients to take action on their own EHR data for sharing with full access control. The system will not only allow individual patients to stay at the center of their care but also enable medical practitioners and researchers to have fast, secure data access to enhance cancer treatment with significantly reduced cost and improved efficiency.

## Methods

### Background on Blockchain Technology

Blockchain is a peer-to-peer distributed ledger technology that provides a shared, immutable, and transparent append-only register of all the transactions that happen in the network [8]. Data in the form of transactions, digitally signed and broadcasted by the participants, are grouped into blocks in chronological order and timestamped. A hash function is applied to the content of the block and forms a unique block identifier, which is stored in the subsequent block, thus forming a “chain.” Due to the properties of the hash function (the result is deterministic and cannot be reversed), one can easily verify if the block was modified by hashing the block content and comparing it with its identifier. The hash of the previous block, as a part of the block content, allows one to ensure the block belongs to its “location.” The blockchain is replicated and maintained by every participant. With this decentralized approach, there is no need to set up a single trusted centralized entity for managing the registry. The participants (in particular, in the permissioned settings) will notice a malicious attempt to tamper with the information stored in the registry; hence, the immutability of the ledger is guaranteed.

Blockchain technology relies on public key infrastructure (PKI) and employs cryptographic primitives such as digital signatures and asymmetric encryption. In case of an asymmetric encryption scheme [16], two keys are generated: One of the keys is publicly known (public key), and the other (private, or secret, key) is kept private by its owner. To send a secret message, the sender encrypts the message with the recipient's public key and sends it. The recipient can decrypt it using his private key. A digital signature is a construct that authenticates both the origin and contents of a message in a provable manner. A user signs the message with his private key, and other users can check the signature with the public key of the signer [17]. PKI is a framework for managing the creation, distribution, identification, authentication, and revocation of public keys [18]. Adding a new block to the existing ledger is defined by the consensus protocol employed in the implementation of the blockchain technology. A consensus protocol is defined as a protocol employed to disseminate requests among the nodes, such that each node executes the same sequence of requests on its instance of the service [19]. Based on the membership mechanism (ie, how the identity of the participant and their right to participate in the consensus are defined within a network, such as proof of work or endorsement policy), one could distinguish between permissionless and permissioned blockchain systems [20]. The role of the proof of work is to define who will be adding the next block (something that is defined at the policy level in case of permissioned blockchains where identities of the participants are known). Permissionless and permissioned systems also usually employ different consensus protocols (eg, Nakamoto consensus, Practical Byzantine Fault Tolerance [PBFT]). Nakamoto consensus realizes a replicated state machine abstraction, where nodes in a permissionless network reach agreement about a set of committed transactions as well as their ordering [21]. The protocol relies on chaining blocks of transactions. Nodes express their acceptance of the block by working on creating the next block in the chain, using the hash of the accepted block as the previous hash. In case of PBFT or other protocols used in permissioned blockchains, the designated set of nodes verifies the validity of transactions based on the credentials of the transaction’s origin. The architecture design of permissioned blockchain technology provides privacy and security guarantees that are impossible to achieve in the permissionless settings.

We restrict our work by following the settings of the permissioned blockchain technology for highly sensitive EHR [22]. A permissioned blockchain network is operated by known entities such as stakeholders of a given industry. Thus, this design choice enables improved control of the participating network and registration of users and minimizes the computational power compared to the expensive proof of work process used in a permissionless blockchain. As a result, better transaction throughput can be achieved while providing improved trustworthiness and preserving the privacy and audit trails of data sharing.

Hyperledger Fabric [23,24] — an implementation of a permissioned blockchain — is an open-source blockchain initiative hosted by the Linux Foundation. Hyperledger Fabric contains a security infrastructure for authentication and authorization (membership service [MS], employing a certificate authority [CA], which is an entity that can generate certificates for key pairs for signing and encryption for the peer nodes and solution users [SUs]). The goal of an MS is to support enrollment and transaction authorization of peers and users through public-key certificates. This is one of the main differences from the permissionless blockchain framework. Hyperledger Fabric also provides the support of anonymous credentials with multiple CAs and the use of threshold signatures. In addition to the MS, the other main architectural components are peers and an ordering-service node, or orderer. Orderer is a node (or a cluster of nodes) running the communication service that implements delivery guarantee, such as atomic or total order broadcast. This is done by transaction verification and ordering. During the verification phase, the digital signature of the transaction issuer is verified, as well as the so-called endorsement policy. The endorsement policy is defined for a chaincode and is used to instruct a peer on how to decide whether a transaction is valid. An example of such a policy can be defined as a requirement that all the peers in the network have to validate (and therefore sign) the transaction. Then, the orderer, during the verification, must ensure that the transaction is indeed signed by all the peers and that the signatures are valid.

In Hyperledger Fabric, smart contracts are implemented by the chaincode. The chaincode is defined by its logic and associated world state (state). The chaincode logic is a set of rules that define how the transactions will be executed and how the state will change. The logic can be written using general-purpose programming language. The state is a database that stores the information in a form of key-value pairs, where the value is an arbitrary byte array. The state also contains the block number to which it corresponds. The ledger manages the blockchain by including an efficient cryptographic hash of the state when appending a block. This enables efficient synchronization if a node was temporary offline, minimizing the amount of stored data at the node.

### System Model of ACTION-EHR

**Figure 1:**
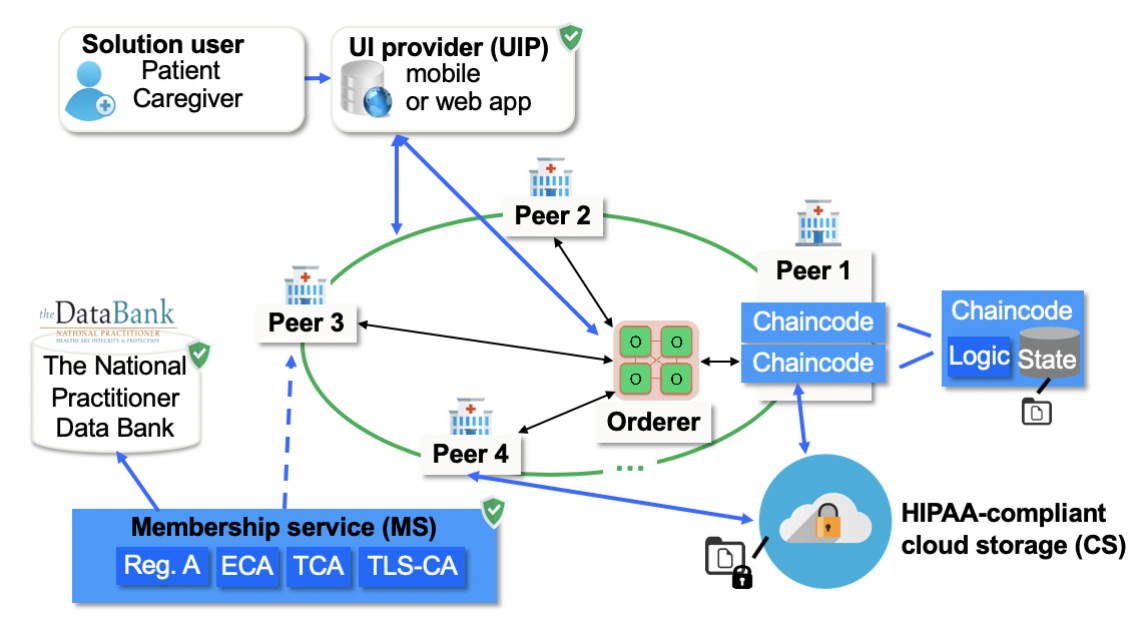
The system model for ACTION-EHR, a distributed patient-centric blockchain-based electronic health record data sharing system based on permissioned blockchain technology and implemented using Hyperledger Fabric v1.4. ECA: enrollment certificate authority; HIPAA: Health Insurance Portability and Accountability Act; TCA: transaction certificate authority; TLS-CA: transport layer security certificate authority; UI: user interface.

ACTION-EHR (patient-centric, blockchain-based EHR management) is a permissioned blockchain-based system for EHR data sharing and integration. Each hospital will provide a blockchain node integrated with its own EHR system to form the blockchain network, and a web application will be used by patients and doctors to initiate EHR sharing transactions. To achieve scalability for the EHR data, ACTION-EHR takes a hybrid data management approach, where metadata on data sharing will be stored on the chain and shared EHR data will be encrypted and stored off-chain in HIPAA-compliant cloud-based storage. A patient (or his health care proxy) will be able to initiate a record-sharing request and define sharing permissions, thus having full control of the shared data. PKI-based [25] asymmetric encryption (that distinguishes between encryption and decryption keys) and digital signatures are employed to secure shared EHR data. The system model of ACTION-EHR framework is shown in Figure 1, and a prototype was implemented using Hyperledger Fabric v1.4.

ACTION-EHR consists of the following components: peer node, SU, client-server web application, MS, orderer, and HIPAA-compliant cloud storage (CS).

**Figure 2:**
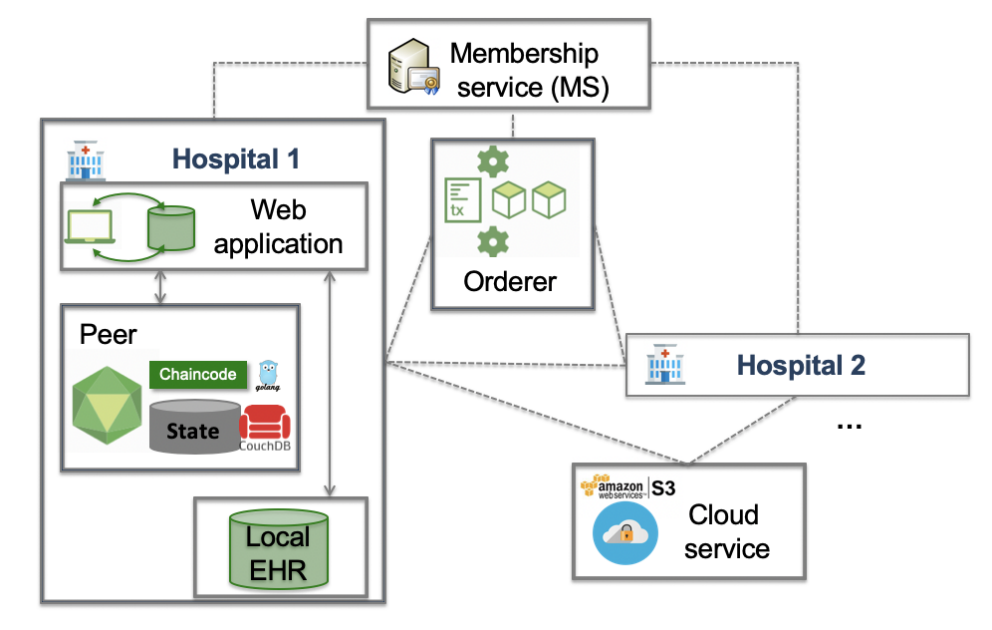
Communication between the components of the ACTION-EHR system (single organization). EHR: electronic health record.

The peer node (1-n) is a peer in the EHR blockchain network representing a health care institution. As each hospital has its own EHR system, the peer node will have access to the EHR system for pulling data for sharing. The peer node has a web server, EHR integration layer, chaincode defining the sharing operations, and a database (ie, CouchDB [26]) for on-chain data management (Figure 2). The peers also agree on the validity of the transactions and maintain the current state of the blockchain ledger by adding new blocks of transactions and updating on-chain data accordingly. Metadata are stored on-chain and consist of EHR metadata (eg, data source, data category) and permission metadata for each EHR record to be shared.

The SU is an end user of the system. Currently, there are 2 SU roles available: patient and caregiver. We also assume that at every hospital, there is a trusted user with a role of administrator for registering new users. The user management will be automatized once the system is integrated into the clinical data flow, and the electronic identity management system in a hospital (ie, ID cards) can be used. The identity of the administrator, as well as the identities of the users, is maintained by a CA.

A client-server web application is used for SUs, who will access the system through a web client (user interface provider). A web server is deployed on each peer node, which interacts with the chaincode. A hospital administrator (Admin) enrolls the users and retrieves the data from the local EHR database. To ensure that the software is trustworthy, the source code can be digitally signed and made available as open source for verification.

The MS is an entity that manages the network identities of all member organizations and users but does not have access to the EHR data or metadata stored on the blockchain. Before registering a peer, the MS uses a trusted database (such as the National Practitioner Data Bank) to verify the peer. To relax the assumptions and provide stronger security and distributed trust, collective authority servers could substitute for a single MS [27]. We assume that the MS is trusted and hosts a standard CA that can generate certificates for key pairs for signing and encryption for the peer nodes and SUs.

The orderer is a service that provides the verification and ordering of the transactions.

The HIPAA-compliant CS is a server where highly sensitive health care data in an encrypted form are stored according to the access-control policy specified by the patient. The CS is used to support exchange of large files such as medical images and can also be employed when constructing the full history of the patient data.

Based on this design, Action-EHR provides the following two user scenarios for the patient and caregiver, respectively.

A patient can log in into the web platform using a web application. The patient can then share his health care data (using automatic fetching via a “record sharing service” responsible for verification of the patient’s existence, the data existence, and pulling the data) from the local database, emulating the hospital database management system with registered caregivers from hospitals that form the blockchain network. To share the data with a caregiver, the patient will need to specify the caregiver, the category of the data to be shared, and for which period of time this caregiver will be able to access the data. The transaction is generated automatically based on the information provided by the patient via the web application and is broadcasted in the network. Simultaneously, prior to being uploaded to the cloud, the data are encrypted. The corresponding transactions that define the metadata of the uploaded data are then added to the ledger.

A caregiver can log into the system and query the ledger to view the permissions specified by the patient, download from the CS with respect to the permissions, and decrypt the data. The patient can revoke the permission given earlier to the doctor by updating the ledger with the corresponding transaction. The patient can also retrieve all historical transactions from the blockchain in chronological order. This can be also used for auditing purposes. Permissions can also be indirectly used to delete the data corresponding to the patient. If the patient wants to delete his data from the CS, he can modify the permissions on the blockchain accordingly. Implementation of the data-deletion process is the next step in our future work.

### Implementation Considerations

#### Cloud Storage

One major challenge for sharing EHR data over blockchain is scalability, as EHR data such as images can be large. Due to the distributed replicated nature of a blockchain network, storing and replicating EHR data on the network for sharing are infeasible, as the large data volume will significantly slow performance. Instead, we propose a hybrid data management approach: All metadata (such as transactions, metadata of EHR, access control) are stored on the chain, but shared sensitive EHR data are stored and managed in a HIPAA-compliant cloud. We adopted Amazon Web Services (AWS), which provides HIPAA compliance through the “AWS Business Associate Addendum” [28]. The shared EHR will be encrypted and stored in AWS storage, which provides high scalability, high availability, and low latency.

#### Blockchain Nodes

The components of the Hyperledger Fabric are provided in the form of virtual containers — a standard unit of software that packages code and all its dependencies. However, in a real-work scenario, each peer will be physically located on the hospital premises; thus, we have to be able to run each peer on a separate machine. For metadata management on-chain, we take a key-value approach, where the “key” is a pseudonym of the patient (that can be generated as a randomly selected combination of letters and numbers or using a hash function), and the value represents the metadata represented in a JavaScript Object Notation document stored in a chaincode state database CouchDB [26], a document-oriented NoSQL database provided by Hyperledger.

#### Web Portal

To interact with the chaincode and manage the users (patients and caregivers), a web portal and set of methods that allow communication between the user interface and the server (chaincode) are required. For testing purpose, we created a simulated EHR database with example patients, EHR data, and caregivers for each node. The web portal of the applications makes asynchronous calls to the representational state transfer application programming interfaces implemented in Javascript. The technologies used to implement the web portal are HTML, cascading style sheets, and Javascript, as well as open-source Bootstrap libraries.

#### Cryptographic Operations

Following best practices for applied cryptography, we ensured that all the SUs possess 2 different key pairs for signing and encryption. The keys are generated during the registration phase. With his secret key for signing (SK_SU_), the SU signs every transaction when exploiting the functionality of the chaincode. Users can verify the authenticity of the transactions and permissions by verifying the digital signature. A hybrid cryptosystem (ie, a cryptosystem that combines the convenience of a public-key cryptosystem with the efficiency of a symmetric-key cryptosystem [29]) is used to encrypt the patient’s data. Patient data are being encrypted with the symmetric key before being uploaded to the CS. Then, the symmetric key is encrypted with the public key of a patient for storage and with the public key of the doctor, with whom the patient wishes to share the data. To decrypt the patient's data, the doctor first uses his corresponding private key to decrypt the symmetric key and then uses it for data decryption. Different approaches can be chosen depending on the available mechanisms to manage the symmetric key. One solution is to use only one patient-specific symmetric key to encrypt the data of this patient and ensure strong protection of this key. Although this theoretically gives the doctors with whom the patient shared the data the opportunity to decrypt all the patient’s data, permissions stored on the ledger strictly manage access to the patient’s data, only allowing the doctors to download the data according to the patient’s access control policy. An advantage is that if a patient wants to share the same data with multiple doctors, he only needs to upload the encrypted data once, and only the key will be shared multiple times. Yet, if such a patient-specific key is compromised, a set of actions have to be immediately taken by the patient to prevent violation of his privacy and restore data availability. Using a newly generated symmetric key for each data-sharing operation will minimize this privacy threat in case of a compromised key yet will require establishment of comprehensive key management and duplication of the patient’s data when shared with different doctors. While both approaches are viable, for the prototype, we used the latter and assumed that the keys are encrypted and managed off-chain and can be stored using existing conventional approaches (eg, smart cards, security tokens, or cloud-based hardware security modules [30]). The public-key encryption ensures confidentiality of the symmetric keys, the symmetric encryption ensures confidentiality of the patient’s data, and the digital signature ensures integrity, non-repudiation (ie, provides proof of the origin), and authenticity.

The properties of the blockchain technology, architecture design, implementation approaches, and cryptographic interfaces guarantee the protection of the sensitive data that flow in the system. These include the following privacy and security properties: data integrity, confidentiality, authenticity, and availability according to the access-control policy, as well as unlinkability between system metadata and the corresponding patient's identity for any unauthorized user (ie, only the users authorized by the patient are permitted to link the patient's identity and his record stored on the blockchain).

## Results

In this section, we present our solution prototype that demonstrates the feasibility of the approach. We describe the data model and the data sharing transaction that are in-line with the system model and required functionalities defined in the section, System Model of ACTION-EHR.

### Overview of the Prototype

To present ACTION-EHR, we use the example of sharing EHR data between oncology information systems for radiation oncology. The EHR data include radiation, medical, and surgical information to assist radiation oncologists and medical physicists to manage different types of medical data, develop oncology-specific care plans, and monitor radiation doses for patients. We also describe one-organization and multiple-organization settings, both of which have been implemented.

Figure 2 shows the one-organization settings: The EHR blockchain network is formed by a cloud server, MS, orderer, and peer nodes (health care institutions; Figure 2). Every hospital participating in the network needs to deploy a server running a Hyperledger Fabric v1.4 peer, interfacing an EHR database (for testing, a simulated EHR database using MySQL was used per peer), an instance of CouchDB for on-chain metadata management, and a web application to interact with the chaincode and EHR.

However, while such approach is easier to set up and maintain, it may not be the best fit in practice: The MS is then required to manage the identities of all the users from different hospitals. Moreover, a certain level of centralization is unavoidable, as separate designated entities are required to host the MS and the orderer.

**Figure 3:**
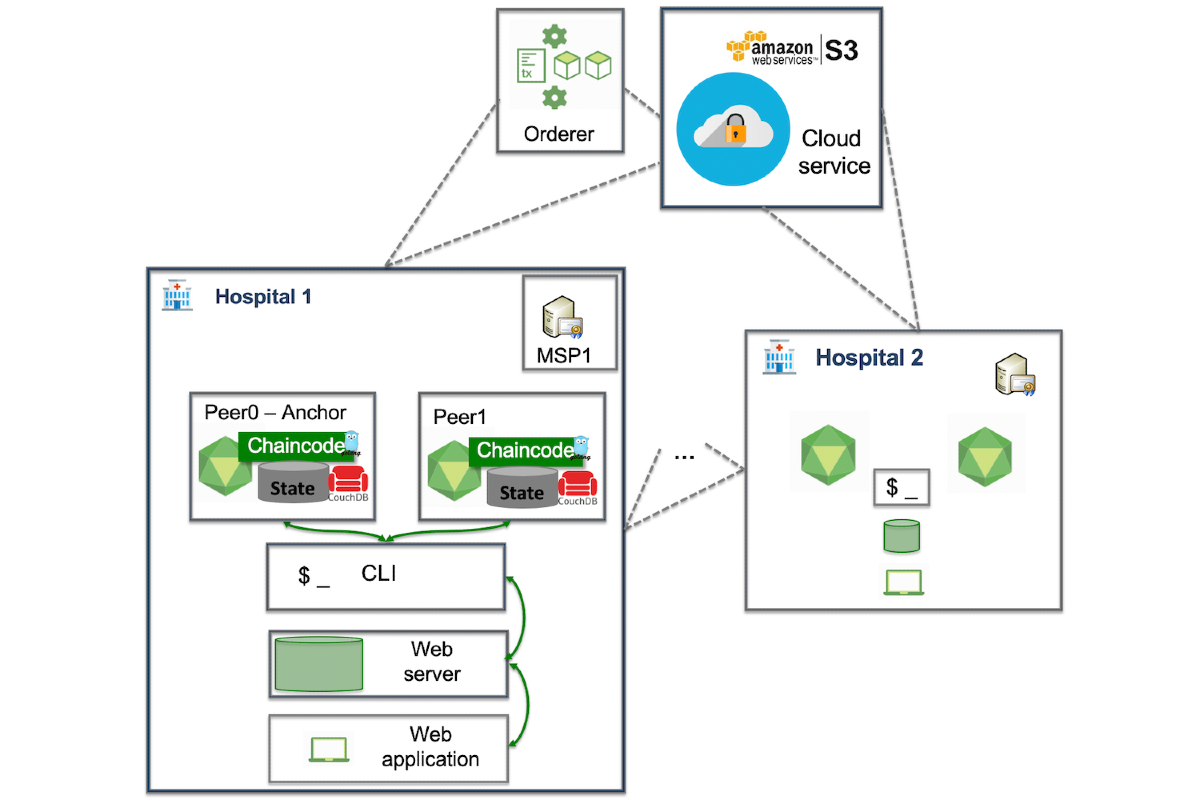
Communication between the components of the ACTION-EHR system (multiple organizations). CLI: command line interface; MSP: membership service provider.

In order to address this issue and provide better levels of decentralization and trust, we employed the concept of organizations from Hyperledger Fabric to create a network of hospitals (as shown in Figure 3), where each hospital will be represented as an independent organization and will have its own MS, orderer, and set of peers and will host a web application. In such settings, user management is distributed, yet a patient is able to choose a hospital and doctor to add corresponding permissions. The network is dynamic: A hospital can join the network according to the policy set up in the network and after exchanging the public part of the cryptomaterials.

### Data Model of Action-EHR

**Figure 4:**
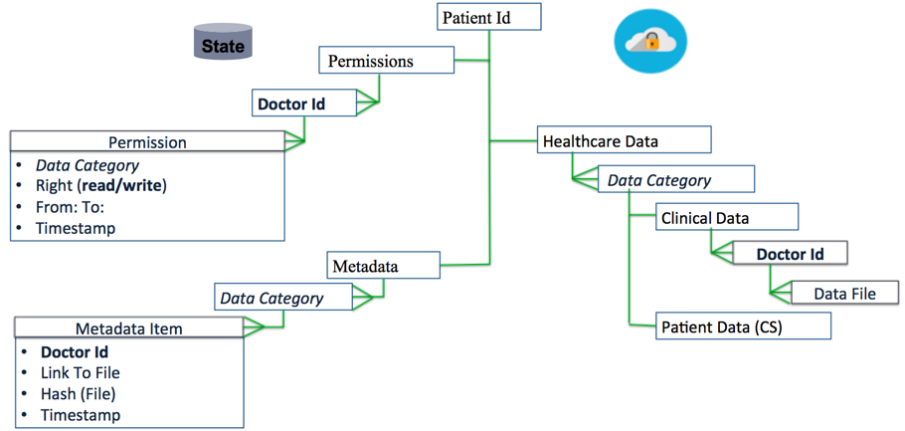
The data structure of the metadata and electronic health record (EHR) data stored on the blockchain and cloud server, respectively. CS: cloud storage.

Figure 4 presents the data model of ACTION-EHR, representing the data structure of EHR data and metadata that are stored on the chaincode and cloud server, respectively. Records on the blockchain are stored in a “key-value” form; “key” is a pseudonym of the patient, and a corresponding patient’s record in JavaScript Object Notation format is stored as a byte array forming a “value” part of a chaincode state.

#### Access Control Metadata

The block containing the information about the permissions is organized as follows. Each permission corresponds to an ID, with which a caregiver is registered in the system. Every permission specifies the timeframe (“from: to:”) during which the clinician has the right to “read the patient's data that fall into a specific ‘data category’ and to upload the data to the cloud repository (“write”).” “Timestamp” enables the patient to update and track access control changes. For patient P to revoke the right for caregiver C to access a specific type of data provided by other caregivers, patient P has to add a new permission with another time frame. To do so, the patient needs to update the ledger by sending the corresponding transaction.

#### EHR Metadata

Clinical metadata is a block that contains information about all the data files uploaded to the cloud by the clinicians or the patient himself. The metadata items are categorized based on the semantics of the corresponding data files. Every item contains an ID for the clinician that uploaded the data (“doctor ID”) or a patient's pseudonym, a pointer to the file that is stored in the cloud (“path to file”) and the hash of the data file (“hash(file)”) to ensure unforgeability of the data stored in the cloud, and the “timestamp” of the moment when the data file was uploaded. It is not necessary to use a digital signature for the file instead of the hash, as the entire content of the transaction that contains doctor ID and hash(file) is digitally signed by the doctor that uploads the file.

### Web Portal for Solution Users

The web application is an implementation of a user interface that provides users easy access to the functionalities of our prototype. There are 3 views of the web portal: administrator (to be merged with the identity management system in a hospital once the prototype is fully integrated into the clinical dataflow), patients (user), and caregivers (user).

The administrator page shows the list of all the patients and doctors from the hospital that are registered in the system. It is possible to enroll a new user via the administrator page by invoking the MS and verifying that the credentials were generated for this user. Through this page, it is also possible to customize the interface (eg, to add or remove a new department or new roles; extending the functionality by adding new roles such as nurse or laboratory scientist is planned for the future development of ACTION-EHR).

**Figure 5:**
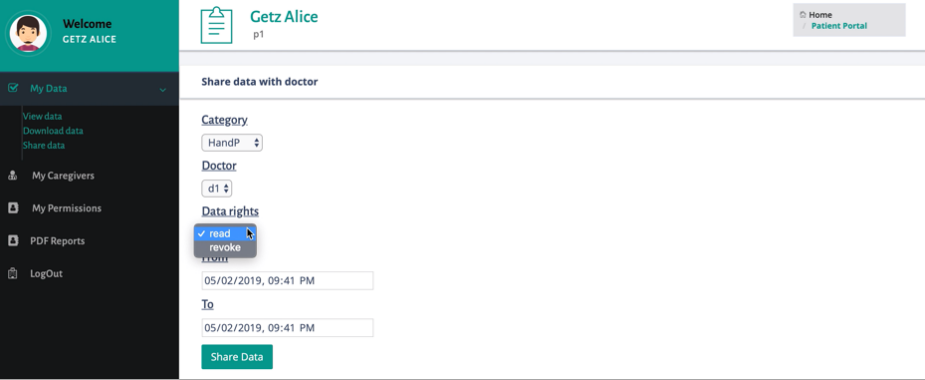
Web portal (patient view): p1 can add a new permission for caregiver d1 (“read” the data for the specified time interval and immediately “revoke” the permission given to the specified caregiver).

When the patient logs in to the patient portal, he can access the patient-specific functionalities of the prototype (Figure 5). The patient can view his data that are currently stored on the cloud, together with the corresponding permissions, including which doctor(s) can access the data and during which period of time. When the patient adds a permission to allow the doctor access the data, the data are encrypted with the public key of this doctor and uploaded to the cloud. The patient can also download his data and modify the access control policy by adding new permissions. The patient can also see the caregivers from whom he is receiving care and a history of all the sharing transactions.

**Figure 6:**
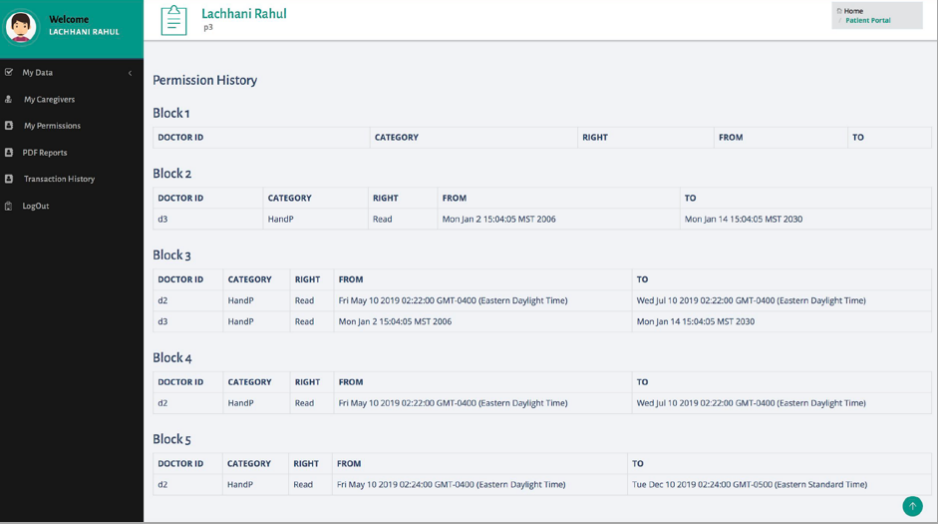
Permission history of a specific patient (p3).

Figure 6 shows the history of all the permissions given by a specific patient. This information can be used by the patient to review his access control policy, as well as for audit purposes. In further development of the system, this information will also contribute to building the full history of the patient’s data.

**Figure 7:**
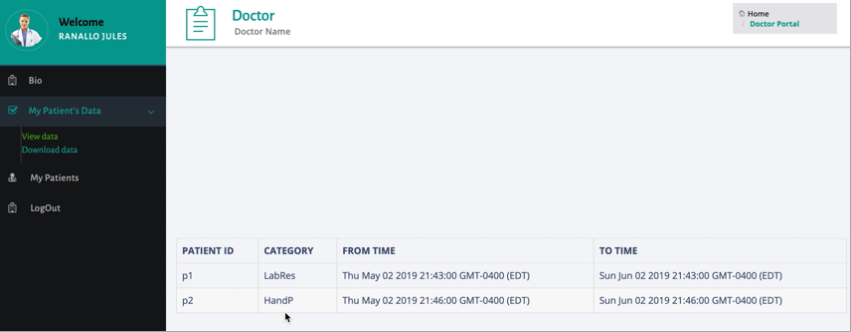
Web portal for the caregiver: view data.

**Figure 8:**
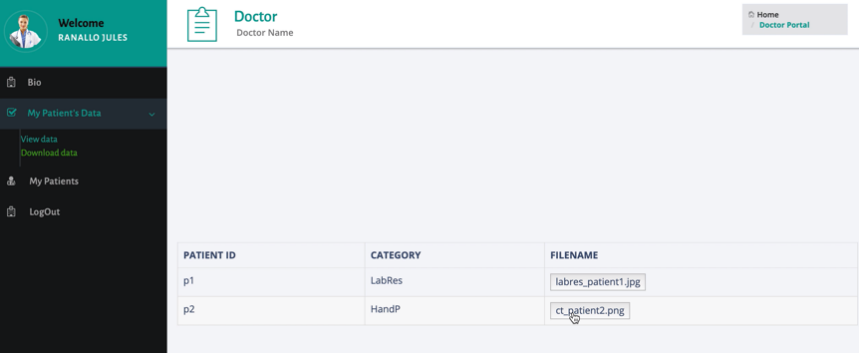
Web portal for the caregiver: download data.

The portal for caregivers (as shown in Figures 7 and 8) allows the doctors to view information for the patients receiving treatment and what data they have shared. The doctor can see which patient shared with him which type of data and during which period of time (Figure 7) as well as download the corresponding patient data files based on the permissions specified by the patient (Figure 8).

## Discussion

### Principal Findings

To achieve the required functionalities of ACTION-EHR (see System Model of ACTION-EHR), we designed the system architecture and data structure of on-chain and off-chain storage. We defined and implemented the data-sharing protocol with respect to the health care scenario and developed the chaincode accordingly. We created the web application that serves as a user interface and enables interaction with the chaincode. To ensure interoperability and seamless integration of our system into the clinical dataflow, we implemented an independent pluggable module that provides conformance with the Fast Healthcare Interoperability Resources (FHIR) standard.

Given the health care environment for which our system has been developed, it is of high importance to ensure security and privacy of multiple data types of different sensitivity levels. Our system guarantees privacy rights and security properties (data integrity, availability, confidentiality, authenticity, and unlinkability) for the following types of data: EHR data, metadata (including permissions or access control policy), and cryptographic keys and user credentials.

Multimedia Appendix 1 contains a detailed description of the threat model, definition of the security properties, and security analysis.

### Limitations

It is challenging to apply a relatively new technology that is not yet framed by government rules and regulations in a highly regulated health care environment. Some technical limitations of our prototype, especially related to the application domain, are highlighted in the paragraphs that follow.

The risk of having a single point of failure of the system can occur if the deployment of the system is not done correctly (if only a single orderer and single CA are employed). Using Kafka cluster and multiple CAs (such as in [27]) can address this limitation. The properties of the group signatures [31] and anonymous credentials [32] could also be explored to address this limitation in future work.

In the health care domain, emergency situations occur regularly, and data might be required urgently. If an unconscious patient arrives at a medical institution and the access control policy is defined such that that no caregiver from the medical institution has a right to access the patient’s EHR, it is impossible to update the permissions and grant caregiver access to the data. Robust and secure “break-glass” mechanisms for emergency situations are required to address this limitation.

According to the new General Data Protection Regulation in Europe, a patient has “the right to be forgotten.” This right might not be easily compatible with the immutability principles of the current implementations of the blockchain technology used in this work; the patient cannot delete his metadata record from the ledger. Applying different cryptographic techniques such as asymmetric encryption, threshold encryption, and proxy re-encryption, as well as principles of redactable blockchains (as proposed in [33,34]) could be used to address such limitations and will require further investigation.

### Comparison With Prior Work

In this section, we describe recent related work employing blockchain technology to achieve fast, secure, and privacy-preserving sharing of EHR. We also underline the differences with our work described in this paper.

A recent review [15] provided an extensive list of studies and ongoing projects that focus on exchanging patient care data using blockchains to improve medical record management, conduct clinical studies, and support health care financing tasks. The authors describe key benefits of using blockchain technology in health care and discuss potential problems and challenges to be considered when adopting permissionless blockchain technology (eg, speed and scalability, confidentiality, the threat of a 51% attack, management of the transaction fees, and “mining”).

The two most mature prototypes are MedRec and FHIRchain. MedRec [13] is a system based on Ethereum smart contracts for an intelligent representation of existing medical records that are stored within individual nodes on the network. The authors propose two incentivizing models for “mining,” including the possibility of accessing the data for research purposes. However, the authors did not propose a mechanism for generation of such anonymous data for research in a decentralized manner while ensuring patient privacy. FHIRchain [10] is a blockchain-based approach for data sharing that encapsulates the HL7 FHIR standard for clinical data. Zhang et al [10] describe a rigorous and deep study of using blockchain technology to transfer EHR. As these two systems use the permissionless blockchain technology, they both face most of the challenges listed by Kuo et al [15] and those already mentioned. Moreover, for instance, in MedRec, the pseudonymous property of transactions and use of a public key as a node address in a current prototype implementation can lead to inferring patterns of treatment from frequency analysis. Even without disclosure of name or personally identifiable information (encryption of the on-chain data and traffic), through the analysis of network communications, one could infer that some interactions have taken place. In our work, we employed permissioned blockchain technology, where only verified nodes are allowed to have access to the ledger. This prevents malicious traffic monitoring. Moreover, we employed encryption of the off-chain data and stored them in the cloud to protect against accidental or malicious violation of confidentiality and unavailability of the patient’s health care data. At the same time, we removed from the original data sources the threats posed when publicly exposing interfaces for data access based on the pointers to the original data sources. In addition, employing an MS allows more flexibility of the user management processes, including rigorous verification of a new user. In our work, we also employed a pseudonymization approach that allows retrieval of the sharing history of a specific patient. In cases of the FHIR chain and other Ethereum-based implementations, public keys are employed as a form of digital identity. However, if the user loses his private key, it is impossible to authenticate this user.

In the space of applying permissioned blockchain technology in health care, the following studies share some similarities with our approach. The work presented by Liang et al [9] focuses on collecting medical data from wearable health devices, such as watches and bands. The authors proposed using the permissioned blockchain technology and storing health care data on-chain. Liang et al [9] implemented an access control scheme by utilizing the MS component and data separation via channels to protect privacy. Our approach is different, as we propose using the blockchain ledger to mainly store the metadata and permissions corresponding to the health care data, which are stored on the cloud service in encrypted form. This enables a more granular access control policy, enhances the data security and privacy, and avoids unnecessary replication of health care data. Magyar [35] used the basic principles of the HIPAA regulation and suggested a list of cryptographic tools that can be potentially applied to ensure data privacy and security, as well as potential approaches to modeling EHR blockchain–based EHR applications. While providing important insights, the work of Magyar [35] is only theoretical; no implementation is provided.

Analysis of the challenges that need to be addressed in the health care industry, as well as the potential benefits of employing blockchain technology, especially a permissioned implementation, can be found in the studies by Krawiec et al [11], Paranjape et al [36], and in a whitepaper from IBM [12]. Our work, while agreeing with the general statements, also focuses on the practical example and extends them by providing analysis of the privacy and security, as well as current limitations and approaches to address them, of a specific implementation.

Peterson et al [14] presented another system design based on the permissioned blockchain implementation (MultiChain [37]) and discussed how FHIR integration into such a system can address the interoperability issue. The proof of interoperability proposed by Peterson et al [14] is based on conformance to the FHIR protocol, which requires verification that the messages sent to the blockchain can get converted to other required formats. This work by Peterson et al focuses on data storage and data interoperability but is limited in terms of the smart-contract functionality that is not supported by the chosen underlying blockchain technology implementation. In contrast, we leveraged the smart-contract functionality to enable a dynamic access control policy definition and to ensure some of the privacy and security properties of our prototype.

Storing data on blockchain can restrict the data volumes that can be efficiently managed and can violate the rights of the patient (ie, to delete data or withdraw from participation in a research study). Motohashi et al [38] described a system design for a blockchain-based system for clinical trials that requires data aggregation from mobile devices. The authors proposed using multiple relay servers to encrypt the data before uploading it to the blockchain. While using relays helps against tampering with and takes on the complexity of data encryption on the mobile device, the relay servers (or at least the majority of them) have to be trusted. This can be acceptable only for anonymized data and if it is impossible to link the data to the real identity of a user or owner of a mobile device. Li et al [39] presented a system to share encrypted prescriptions data and used the same underlying implementation of private permissioned blockchain technology and, similar to our work, key-sharing mechanism. However, as in the study by Motohashi et al [38], Li et al [39] chose to store the data on the blockchain. In ACTION-EHR, only metadata are stored on the blockchain. Hylock et al [40] presented a patient-centric blockchain framework that supports a set of configurations (different modes related to the encryption and data-storage modes). To comply with legislation, the authors proposed an alternative approach: to use redactable blockchain [41] to build the ledger that consists of immutable and non-immutable blocks. The authors however do not provide a multinode implementation, which makes it impossible to evaluate proof-of-concept presented in the paper. The authors also proposed storing the data at the original data sources, which, as already discussed, can introduce data unavailability and security threats.

Pournaghi et al [42] proposed using both permissioned and permissionless blockchain technologies, the former to exchange the pointers to the encrypted data stored in the cloud, as well as the symmetric encryption key, encrypted with an attribute-based encryption scheme. The latter is used to distribute the description and access-control structure for the data stored in the cloud. The authors proposed using PBFT-based consensus for both blockchains, which can introduce scalability issues. In addition, defining an access structure for an attribute-based encryption scheme requires specification of the attributes for medical and patient profiles, using a format of the tree where each inner node is a threshold gate and the leaves are the attributes. It can be difficult for a patient to construct such an access-control structure. Sharing such a structure, as well as sharing cryptographic keys (even in the encrypted form) on the blockchain, could also create a threat to patient privacy. While we use a similar concept of storing encrypted data on the cloud and sharing pointers on the blockchain, we propose exchanging the keys off-chain and enforcing an access-control policy by letting the cloud verify the consistency of the ledger prior to sharing the data. We propose a simpler yet secure approach for defining an access-control policy; permissions are defined for pseudonymized users and are stored on a private permissioned blockchain.

A plethora of existing blockchain platforms and various prototypes built on top of the technologies can aggravate the lack of interoperability between health care systems that is highly relevant due to multitude EHR systems with different interfaces. Thus, ensuring interoperability between different blockchain platforms is of high importance and shall be considered as one of the possible directions for future work. Moreover, due to custom privacy requirements and individual needs of different patients, one can think of a multiple-ledger design: a patient-specific or even case-specific ledger [43]. Data then can be replicated among multiple ledgers and locations, creating the network of networks [44].

### Conclusions

In health care, a distributed ledger can be seen as a shared immutable and transparent history of all the actions performed by eHealth users; these actions include defining access control policies and sharing, accessing, and modifying the data. This work presents the architecture of the framework for the specific data sharing case for radiation oncology and the implementation of a prototype that ensures privacy, security, availability, and granular access control over highly sensitive patient data. The methodology is general and can be easily extended to support other types of patient care.

The functionality of the prototype meets the requirements from a medical practice perspective. To ease the adoption of the prototype, we implemented an independent pluggable module that conforms with the FHIR standard. Our next step is to set up a pilot network of health care institutions in the United States and Switzerland for further testing of ACTION-EHR with patient data. Once adopted by the health community, such a system will reduce the turnaround time for data sharing, improve decision making for medical care, and reduce the overall cost.

## Appendix

Multimedia Appendix 1 Security analysis.

## Abbreviations

AWS: Amazon Web Services
CA: certificate authority
CS: cloud storage
ECA: enrollment certificate authority
EHR: electronic health record
FHIR: Fast Healthcare Interoperability Resources
HIE: health information exchange
HIPAA: Health Insurance Portability and Accountability Act
MS: membership service
PBFT: Practical Byzantine Fault Tolerance
PKI: public key infrastructure
SU: solution user
TCA: transaction certificate authority
TLS-CA: transport layer security certificate authority
UI: user interface
