# Supplementary material for: ACTION-EHR: Patient-Centric Blockchain-Based Electronic Health Record Data Management for Cancer Care
Source: J Med Internet Res. 2020 Aug 21;22(8):e13598. doi: 10.2196/13598 (PMC7474412; doi:10.2196/13598)
Supplement: Multimedia Appendix 1 [file jmir_v22i8e13598_app1.pdf]

Multimedia Appendix 1. Security Analysis

Multimedia Appendix 1A. Privacy and Security Properties

In what follows, we list and define the privacy and security properties that a blockchain-based sensitive healthcare data-management system shall have. We then present a threat model and provide security analysis, i.e., discuss how security and privacy are guaranteed in our system.

From a philosophical point of view [41, 42], privacy can be defined along three main lines. First, privacy as a person's will to determine which personal information may be communicated to others, second, privacy in relation to a person's control over access to the information about himself, and, third, privacy as limited access to the personal information. Therefore, we could define a patient's privacy as his right to trace and control the personal data that flow between various systems and are shared among different peers.

For the system to comply with the legislation [23], each patient needs to provide consent to share his data both for primary care and research purposes. For the system described in the paper, the following security properties are essential: data integrity, authenticity, availability, data confidentiality, and unlinkability. In the scope of the functionality of our system, the properties are defined as follows:

- Data integrity: the users of the system are ensured that the data were not altered in transit or at rest by an adversary.
- Authenticity: the users are ensured that the data were sent by the claimed sender.
- Availability: the data are available from anywhere at any time, according to the access-control policy specified by the patient.
- Confidentiality: the information disclosure to unauthorized individuals is not possible.
- Unlinkability between system metadata and the corresponding patient's identity for any adversary: only the users authorized by the patient are permitted to link the patient's identity and his record stored on the blockchain.

Multimedia Appendix 1B. Assumptions and Threat Model

For the current implementation, we make the assumptions and define a threat model as follows.

- Membership service manages the identities of users but does not have access to the healthcare data or the system metadata stored on the blockchain. In the current implementation, we assume that the membership service is trusted and cannot be compromised by the adversary. To relax the assumptions and to provide stronger security and distribute trust single membership service could be substituted by Collective Authority servers. An example of a scalable solution is presented in [19].
- We restrict our work by following the settings of the permissioned blockchain technology. In the current prototype implementation, we also use a single certification authority. However, alternative implementations are possible with Hyperledger Fabric, such as support of anonymous credentials with multiple certification authorities and the use of threshold signatures.
- We assume that a Web Application is a trusted software deployed within a clinical infrastructure to construct and forward transactions from the users to the nodes of the network. To ensure that the software is trustworthy, the source code can be digitally signed and be made available as an open source for the verifications.
- We assume that secure communication channels have been established over HTTPS protocols between all parties in the system. Given the medical context, we assume

that medical institutions are committed to providing compliant data-sharing for better patient-care and will not have incentive to behave maliciously but can still be temporarily unavailable. We also assume that peer nodes cannot collude, and no collision is possible between solution users, membership service, and the cloud storage server. Collusion that can happen between multiple peers due to the malware installed can be prevented by setting up the corresponding endorsement policy: f.ex., the transaction will only be accepted, if all the nodes executed it with the same result (and signed it). We further assume that the data from the network layers (e.g., IP address) cannot be used to leak users' identities. This assumption is reasonable, as many users only access the Internet through a NAT (Network Address Translation) gateway offered by their Internet provider, and could be relaxed if, for instance, users and nodes employ a VPN (Virtual Private Network) service or anonymous networks (e.g., Tor [35]) to access the Internet.

We assume that the adversaries can be among users of the system, ADV1, a cloud server ADV2, an outsider, any user that is not registered in the system ADV3, and the nodes, ADV4.

ADV1: Patient (ADV1a) can be an active adversary. For instance, a malicious user could try to impersonate another user and to gain access to another patient's healthcare data, or to learn the system metadata (e.g., permissions set up by the patient for the caregivers). We assume that caregiver, C, (ADV1b) is honest-but-curious, and will not collude with other caregivers: he will use the system to verify his rights, to read the data (provided by the other caregivers and patients) and upload new healthcare data about the patient according to the patient consent. We do not try to protect against false information provided by the caregivers, as we assume that medical doctors do not have incentive for generating false content. We assume that the doctors can not aware of and therefore will not be able to follow the access control policy specified by the patient regarding the healthcare data uploaded by other caregivers and the patient before C uses our system.

ADV2: A Cloud Server, CS, can be an honest-but-curious adversary. It is employed to temporally store shared patient's data. Cloud Server is a HIPAA-compliant cloud storage, i.e., it manages the protected health information (PHI) and ensures that all the required physical, network, and process security measures are in place and followed. We assume that CS will not collude with any other entity. CS will store the encrypted data and provide an access, only according to the access control policy stored on the blockchain (including the deletion of the patient's data after access-control policy is expired). However, CS might try to gain access to the highly sensitive healthcare data.

ADV3: Outsiders, or external observers, could try to get access to the healthcare data, system metadata, or cryptographic keys by trying to impersonate solution users, or to create fake profiles in the system.

ADV4: Blockchain nodes are modeled as Byzantine nodes. Depending on the endorsement policy set up in the implementation, to ensure consensus among the nodes maintaining the blockchain, different number of nodes must be honest for the system to function. Given the application scenario and the medical context, we assume that medical institutions are committed to providing compliant data-sharing for better patient-care and will not have

incentive to behave maliciously but can still be temporarily unavailable. We assume that the nodes will follow the steps required to reach consensus and will maintain the blockchain to ensure that patient's access-control policy is expressed on the ledger and can be accessed by the authorized users.

Multimedia Appendix 1C. Privacy and Security Analysis

Hereafter, we present the privacy and security analysis of the proposed framework in order to show that the privacy and security properties defined above are guaranteed for the following types of data: EHR data, metadata (including permissions or access control policy), and cryptographic keys and user credentials.

Data integrity and authenticity. To ensure data integrity and authenticity, we have to guarantee that the users are ensured that the data were not altered in transit or at rest by an adversary and that the data were sent by the claimed sender. The integrity and authenticity of the EHR data can potentially be violated when the users upload the data to the CS, during the time that the data are being stored on the CS (from the moment they were uploaded by a user till the expiration of the corresponding permissions); and when the data are being downloaded from CS. The integrity and authenticity of the metadata can potentially be violated every time a transaction is issued by a user or the CS and when the peer nodes run consensus protocol to update the ledger. In particular, the integrity and authenticity of the system metadata can be at risk in the following cases: when patient P updates or queries his record on the ledger, when the caregivers verify their permissions on the ledger, when CS queries the nodes, and from the moment system is running and the nodes must maintain the ledger. The threats to the integrity and the authenticity of the cryptographic keys exist during the whole lifecycle of the private keys.

To ensure the authenticity of the healthcare data and the metadata provided by the users, the patients and the caregivers digitally sign all the transactions with their corresponding private keys. To guarantee the authenticity and the integrity of the healthcare data, metadata are stored on the blockchain. Metadata consists of the hash of the corresponding healthcare-data file uploaded to the cloud server and the information about the user that uploaded the data file. When the CS or users queries the ledger, the replies from the peer nodes are signed to ensure data authenticity and integrity. Because we assume that the nodes and the users can securely manage the secret keys and the credentials generated during the enrollment, the integrity and authenticity of the healthcare data and the system metadata are guaranteed by the correctness and unforgeability of the digital signature algorithm and the properties of a secure cryptographic hash function.

The integrity of a patient's record stored on the blockchain is based on the employed consensus protocol that ensures an atomic broadcast and its properties: validity, agreement, integrity, and total order. Private keys of the patients and independent caregivers are stored on the smart card or the mobile phone protected by the pin code known only to its owner. Therefore, no adversary can access and/or tamper the keys at rest. Caregivers store their keys in the secured clinical infrastructure. The keys can only be accessed after a two-factor authentication (e.g., a badge of the medical doctor and his password, received at the registration). If private keys of a user are lost or compromised by an adversary, the fresh keys and certificates will be signed by a membership service, based on the uniquely identifiable information of the users.

Availability. Ideally, the system should guarantee that the data can be accessed from anywhere at any time. However, in our system, we require data availability with respect to the access-control policy specified by the patient. The availability of EHR data can be at risk if the CS is off-line, if the data were deleted or never uploaded to CS, if there is no corresponding permission, and if a user does not possess the corresponding keys to decrypt the data. The availability of metadata cannot be guaranteed if the network of peer nodes is down. The availability of cryptographic keys is at risk if the user loses his credentials and/or his smart card. The EHRs are stored on a HIPAA compliant fault-tolerant cloud storage with respect to the permissions specified by the patients. Thus, the data are erased when the permissions stored on the ledger get expired. We also assume that the patient is able to express his consents/access-control policy by specifying the permissions and sharing their keys, respectively, through the Web portal. The availability of the system metadata via the blockchain is guaranteed by the properties of the atomic broadcast and availability and trustworthiness of the Web applications (UI). The availability of the healthcare data also depends on the availability of cryptographic keys. If a key pair used for the data encryption is lost, the patient will be able to access the permission history, upload the data encrypted with the fresh new public key of the doctor, and update the permissions correspondingly. If the patient loses his login credentials, their recovery can be performed via identity management system in the hospital(s) where patient is registered. Currently, in our prototype implementation, this can be done via hospital administrator Web portal.

Confidentiality. Confidentiality is ensured when the disclosure of information to an unauthorized individual is not possible [24]. The confidentiality of the healthcare data could potentially be violated from the moment the data are sent from the local database of the medical institution/caregiver/patient to the CS. The confidentiality of the data can be violated when the data are in transit, i.e., when being uploaded/downloaded by a solution user, and at rest, i.e., while being stored on CS or on the ledger. The confidentiality of the metadata could be violated if the content of the ledger is revealed to an unauthorized user. The confidentiality of the cryptographic keys can be violated if the user's smart card and credentials are compromised. In our system, the confidentiality of the healthcare data is ensured first by employing the HIPAA compliant cloud storage that will provide access to the data only with respect to the corresponding permissions. Second, confidentiality is ensured by the security properties of the asymmetric encryption algorithm applied to encrypt the patient’s data. To ensure the confidentiality of the system metadata, the system has to guarantee that caregiver C can learn only about the permissions specified for C (and nothing about the permissions specified by P for any other users). In our system, the chaincode implementation guarantees that C can only query the permissions that corresponds to him. The confidentiality of the doctor’s private key is protected by the pin code of the smart card or the phone known only to its owner. If the private key of the doctor is compromised, the patient can add corresponding permissions and request to delete the data. The doctor will need to generate a new key pair; the patient will need to update the permissions and upload the data encrypted with the fresh new public key of the doctor.

Unlinkability. The unlinkability property in our system is defined as impossibility for any unauthorized user that has never been authorized by the patient to link the system metadata and the corresponding patient's identity. This property relies on the identity management approach that we use. When patient is registered in the hospital, he receives a pseudonym

(that is generated as randomly selected combination of letters and numbers). Therefore, only the doctor, with whom the patient has ever shared the data can link the identity and the pseudonym. Cloud service, as well as the nodes-participants of the blockchain network (hospitals) that do not have an authorization to access patients' data, are not able to link the identity of the patient and the pseudonym, thus the system metadata as well. We cannot make a user "forget" the data he already had a legitimate access to. Once a user becomes unauthorized, we can only limit the linkage of the patient's identity with the data. When querying the ledger, the duration of the permissions is taken into account: if the permission is expired, no more metadata will be linked to the identity.
Unless there is a collusion between blockchain nodes (maintained by the hospitals) and the caregivers (and in the system design it is assumed that the collusion is not possible between the caregivers and the blockchain nodes), linking the identity with the record stored on the blockchain is impossible.
